# 

**Published:** 1845-01

**Authors:** 


### CONTENTS

OF THE

### MEDICAL EXAMINER.

NEW SERIES—NO. I.—JANUARY, 1845.

### ORIGINAL COMMUNICATIONS.

Report of Cases of Scarlatina, having relation to the question of the contagiousness of that Disease. By J. K. Mitchell, M. D., Professor of Practice of Medicine in Jefferson Medical College of Philada. - 7

Successful Case of Lithotomy and the Removal of three Calculi, in a boy three years old. By Rufus L. Haywood, M. D., of Tuscaloosa, Ala. 9

Case of Catalepsy, with Convulsions of the right Arm. By John W. Irby, M. D., of Blacks and Whites, Nottoway Co., Virginia, - - 11

On the Cause of Malaria. By Washington L. Atlee, Professor of Chemistry in Pennsylvania College. (Letter to the Editor,) - - 12

### CLINICAL LECTURES AND REPORTS.

PHILADELPHIA HOSPITAL,

*Saturday, December 7, 1844.*

Clinic of Professor Dunglison.

1. Rheumatism, - - - - - - - - - 16
2. Case of Thoracic Disease, - - - - - - - 18
3. Various Pathological Specimens, - - - - - - 19

PENNSYLVANIA HOSPITAL,

*Service of Dr. Randolph.*

Cases admitted into the Surgical Wards of the Hospital since October 20, 1844, (73 cases,) - - - - - - - - - - - 22

WILL'S HOSPITAL (FOR THE BLIND AND LAME.)

*Service of Dr. Hays.*

Thirty-Eight Cases, - - - - - - - - - - 22

### BIBLIOGRAPHICAL NOTICES.

Lectures on the Theory and Practice of Physic. By John Bell, M. D., Fellow of the College of Physicians of Philadelphia: Corresponding Secretary of the Philadelphia Medical College: Member of the American Philosophical Society, and of the Georgofili Society of Florence, etc. etc. And by William Stokes, M. D., Lecturer at the Medical School, Park Street, Dublin; Physician to the Meath Hospital, etc. etc. Third Edition, enlarged and improved. In two volumes. 8vo. 26

Manual of Chemistry. By Lewis C. Beck, M. D., Professor of Chemistry in Rutger's College, New Jersey, and in the Albany Medical College. Fourth Edition, Revised, and Illustrated with numerous wood-cuts, - - - - - - - - - - - - - 29

The Cyclopædia of Practical Medicine. Edited by John Forbes, M. D., Alexander Tweedie, M. D., and John Conolly, M. D. Revised, with additions, by Robley Dunglison, M. D. - - - - - 30

An Introductory Discourse on Medical Education, delivered to the Students of Geneva Medical College, October 1, 1844. By Charles Lee, A. M., M. D., Professor of General Pathology and Materia Medica in Geneva College. Published by the Class, - - - - - 31

### EDITORIAL.

New Series of the Medical Examiner, - - - - - - - - 32

Philadelphia Hospital, Blockley, - - - - - - - - - 33

### RECORD OF MEDICAL SCIENCE.

Annual Election of the Philadelphia Medical Society, - - - - - 33

Laporte University, Indiana, - - - - - - - - - - 34

Board of Naval Surgeons, - - - - - - - - - - - - 34

List of Assistant Surgeons found qualified for promotion, and candidates qualified for admission into the Navy of the U. S. as Assistant Surgeons, - - - - - - - - - - - - - - 34

Vermont Medical Society—Proceedings at the Annual Meeting, Oct. 16, 1844, - - - - - - - - - - - . - - - - 34

Contributions to Therapeutics. By J. Moore Neligan, M. D., Physician to Jervis-street Hospital, Lecturer on Materia Medica and Therapeutics in the Dublin School of Medicine, &c., - - - - - 35

On the Employment of Conium in Painful Diseases, - - 35

Case 1. Obstinate Rheumatic Pains from Exposure to Cold and Wet, - - - - - - - - - - - - - - - 37

Case 2. Severe chronic Arthritis with Swelling and Deformity of the Joints, - - - - - - - - - - - - - - 38

Case 3. Sub-acute Rheumatism confined to the muscular part of the Calves of both Legs, - - - - - - - - - - 39
Case 4. Acute Rheumatism, - - - - - - - - - - 40
Case 5. Chronic Rheumatism caused by constant Exposure to Damp, - - - - - - . - - - - - 41
Case 6. Facial Neuralgia, - - - - - - - - - - 42
Case of Fistulous Communication between the Intestinum Ileum and Urinary Bladder, simulating Stone in the Bladder. By W. C. Worthington, Esq., Fellow of the Royal Medical and Chirurgical Society. Communicated to the Medical Chirurgical Society of London, by James Copland, M. D., F. R. S., - - - - - - - 43
Case of Mollities Ossium, with Observations. By S. Solly, Esq,, F.R.S., Assistant Surgeon to St. Thomas's Hospital, - - - - - 44
Case of Hæmorrhage of the Liver. By Jas. Abercrombie, M. D., Cape of Good Hope, - - - - - - - - - - - - - - 45
Tracheotomy in the advanced stage of Croup. By M. Scoutteten of Strasburg, - - - - - - - - - - - - - - 45
Cure of Nævi by Croton Oil. By M. Lafargue, - - - - - - - 46
On the Re-production of the Crystalline Lens, - - - - - - 46
Treatment of Bed-sores, - - - - - - - - - - - - 46
On the Mechanism or Nature of Atrophy, - - - - - - - - 47
Fatal Case of Glanders, with Remarks. - - - - - - - 47
M. Trousseau on the Signs of Auscultation in Young Children, - - 49
Case of Ulceration of the Duodenum, in which the gall-bladder was filled with a colourless aqueous fluid, and contained numerous gall-stones. By C. J. Roberts, M. D., Physician to the Welsh Charity, &c., - 50
Case in which the Vena Cava Inferior was obstructed from the commencement of the common Iliac veins, and its cavity obliterated between the entrance of the emulgent and hepatic veins. By Thomas Bevill Peacock, M. D., - - - - - - - - - - - - - - 51
Removal of Part of the Base of the Tongue, - - - - - - - 53
Treatment of Chancres, - - - - - - - - - - - - 54
Lardaceous Scirrhoma of the Lung, involving the First Rib, Clavicle, &c., 54
Malformation of the Genitals, - - - - - - - - - - 55
M. Oesterlen on the Passage of Metallic Mercury into the Blood and various Organs of the Body, - - - - - - - - - - 55
M. Ribes on Section of the Flexor Tendons of the Knee in White Swelling, - - - - - - - - - - - - - - - - 56
Spontaneous Rupture of the Inferior Cava, - - - - - - - 57
Analysis of the Blood in a case of Lead Colic. By Professor Cozzi, - 57
Rare case of Milk Cyst in the Breast, under the care of M. Jobert, Hôpital Saint Louis, - - - - - - - - - - - - - - 58
Case of Congenital Deficiency of the Sternum, - - - . - 60
Melancholia Puerperalis Atonicta, - - - - - - - - - 60
Ovarian Dropsy, discharging itself by the Umbilicus, - - - - 60

Amnesia complicating Acute Rheumatism, - - - - - - 61
On Preserving Bodies for Dissection. By John Inglis Nicol, of Inverness, - - - - - - - - - - - - 61
Case of Disease of the Bones and Joints of the Lower Extremity;—Spontaneous Dislocation of the Hip Joint;—Amputation;—Discharge of Calculi from the Bladder;—Recovery. By William Monro, M. D., Surgeon to the Dundee Infirmary, 62
Death from an Overdose of Nitrate of Potass, - - - - - 67
Case of Bi-Partite Vagina; with Spontaneous Rupture of the Septum, and Natural Delivery, - - - - - - . - - 68
Case of Fungus from the Synovial Capsule of the Knee, under the care of M. Gerdy, of the Hôpital de la Charite, treated by Compression, - - - - - - - - - - - 68
Treatment of Hydrocele with Ioduretted Injections, - . - - 69
Case of Death following the Excessive Use of Ardent Spirits; in which Appearances Simulating Irritant Poisoning were found on Dissection. By John Inglis Nicol, M. D. - - - - - - - - - 70
Death of Dr. Abercrombie, - - - - - - - - - - 70

